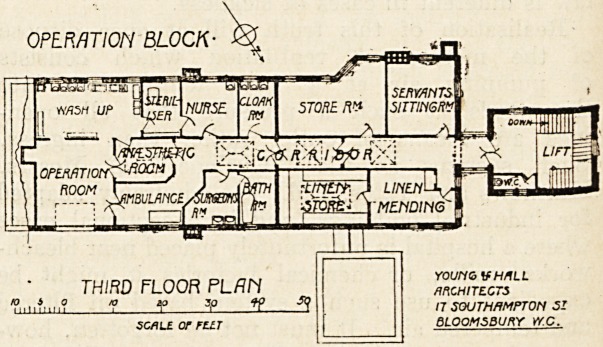# The Miller General Hospital, Greenwich

**Published:** 1911-09-16

**Authors:** 


### THE MILLER GENERAL HOSPITAL, GREENWICH.

The new wing, of which we publish plans to-day, is part of a carefully considered scheme for what will eventually be a large general hospital adapted for the needs of the wide area in South-East London, for which there is practically no hospital provision at present. For obvious reasons it is not desirable to indicate the lines upon which the completed building has been designed. All that can be given at present is the plan of the ward block, and its connection with the existing Miller Hospital.

The building is, including part basement, five storeys high. The basement storey runs under a portion only of the wing; under the remainder of the ward there is an open space 5 feet high, freely ventilated by arches in each side wall. The basement portion contains a mattress store, a sterilising room, a room for dirty linen, together with store rooms for various purposes.

On the ground floor will be the main entrance to the hospital, until the future administration block is built. This leads on the right to the ward wing. Each of the three floors of the ward wing is similar to the others and contains in each instance a large ward for 16 beds, a single bed ward, a ward kitchen, bathroom, testing-room, stores for patients' clothes and linen, and the usual sanitary offices. These latter are placed in a projecting tower just off the entrance end of the ward, and are, therefore, available not only for the patients in the large ward, but for the small ward. This arangement leaves the further end of the ward free, and is in every way more convenient than the plan of putting the sanitary offices in two towers at the further end of the ward. It is clear that the ventilation and lighting of the ward will be infinitely better than it would be with the latter arrangement.

On the sunny side of the ward is a balcony 8 feet wide, with direct access from the ward in two places so that beds can be wheeled out. A staircase at the end of this balcony provides means of escape in case of fire, required by the London Building Act.

The alterations to the existing building on this floor consist of the provision of a suitable office for the matron, also the secretary and his clerks. At present the board-room in the front building serves as a secretary's office and his clerks' office is the room on the ground floor of the hospital now to be used as matron's office. The present entrance to the hospital will be removed, and the space added to the small ward.

On the first and second floors the ward arrangements are precisely the same as those on the ground floor. On the top floor will be found the operation department, consisting of cloak room, nurses' room, sterilising room, bath-room, surgeon's room, anæsthetic room, wash-up room, and operating room. These rooms are all grouped together at the further end of the block. At the near end will be a servants' sitting-room, linen-mending-room and store, and a large room at present not allocated. These arrangements are probably merely temporary.

On the second floor in the old building the kitchen is very considerably enlarged and a larder added, and on the third floor the nurses' dining-room is enlarged and a pantry added.

The works are being carried out by Mr. W. Nash, of Deptford, under the supervision of the architects, Messrs. Young and Hall, of 17 Southampton Street, Bloomsbury.

## Figures and Tables

**Figure f1:**
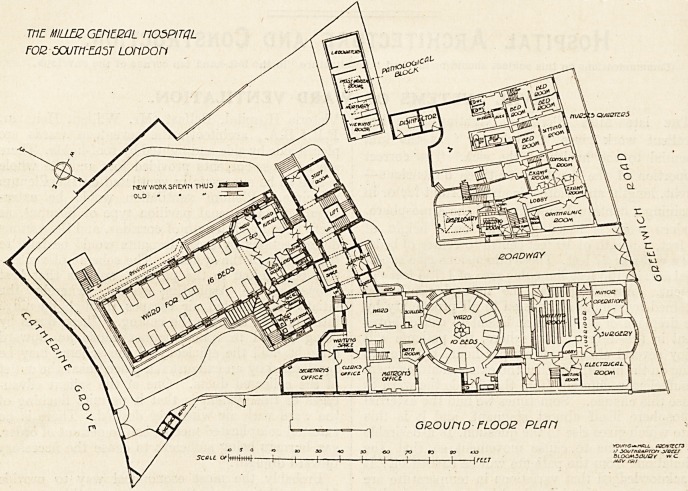


**Figure f2:**